# Supplementary material for: Clip-on lens for scanning tunneling luminescence microscopy
Source: MethodsX. 2024 Jul 2;13:102828. doi: 10.1016/j.mex.2024.102828 (PMC11299554; doi:10.1016/j.mex.2024.102828)
Supplement: Supplementary file 1 [file mmc1.pdf]

Supplementary Material for Article

Clip-on lens for scanning tunneling luminescence microscopy

Authors

Aleš Cahlík[1,,$], Cinja C. Müller[1], Fabian D. Natterer[1]*

Affiliations

[1]Department of Physics, University of Zurich, Winterthurerstrasse 190, CH-8057 Zurich, Switzerland

[$]Present Address: Department of Applied Physics, Aalto University, Aalto, FI-00076 Finland

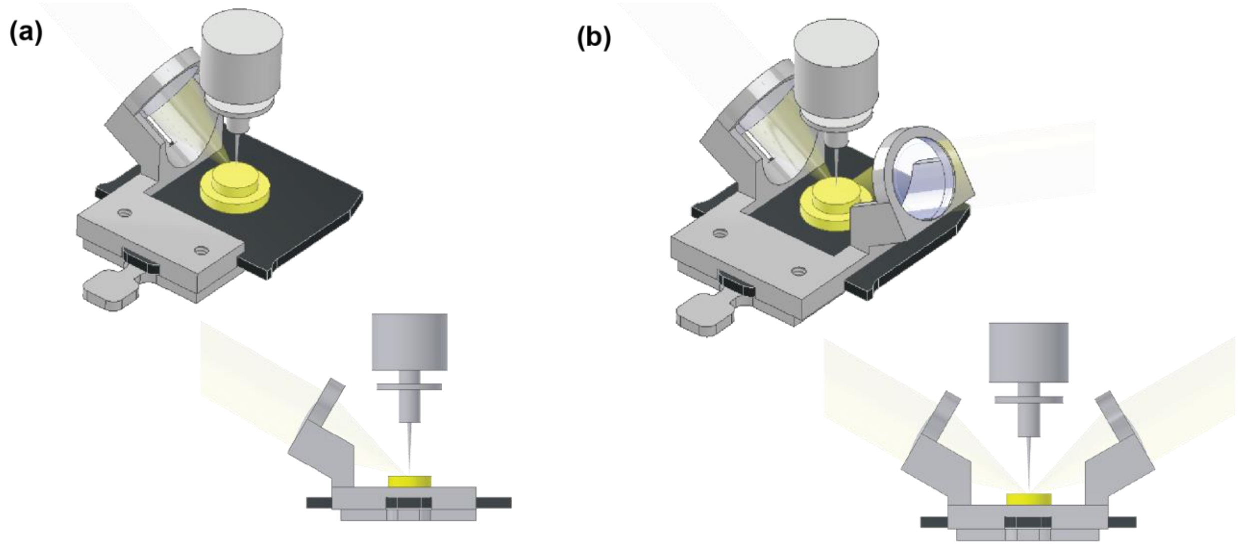

Figure S1: ***Conceptual clip-on lens design for perpendicular optical axis.*** *(a) Lens holder that can be reversibly mounted on the handle of a sample plate, allowing for an optical axis perpendicular to the main sample plate axis. (b) For many existing microscopes, this design would, in principle, allow for collecting from both sides, increasing its efficiency. Note: These proposed concepts were not experimentally tested.*

List of Provided STEP Files

1. **holder_handle.stp**: Common flag-style handle piece used in all concepts.
2. **holder_lens.stp**: Concept validated in the current work for Omicron VT-STM.
3. **holder_lens_pp_1s.stp**: Non-validated concept for a perpendicular optical axis (Fig. S1a).
4. **holder_lens_pp_2s.stp**: Non-validated concept for a perpendicular optical axis (Fig. S1b).
